# Prevalence and Associated Factors of Psychological Distress Among Single Fathers in Japan

**DOI:** 10.2188/jea.JE20210273

**Published:** 2023-06-05

**Authors:** Bibha Dhungel, Tsuguhiko Kato, Yuko Kachi, Manami Ochi, Stuart Gilmour, Kenji Takehara

**Affiliations:** 1 Department of Health Policy, National Centre for Child Health and Development, Tokyo, Japan; 2 Graduate School of Public Health, St. Luke’s International University, Tokyo, Japan; 3 Department of Social Medicine, National Centre for Child Health and Development, Tokyo, Japan; 4 Department of Public Health, Kitasato University School of Medicine, Kanagawa, Japan; 5 School of Humanities and Social Sciences, Tokyo Metropolitan University, Tokyo, Japan; 6 Department of Health and Welfare Services, National Institute of Public Health, Saitama, Japan

**Keywords:** psychological distress, single fathers, non-single fathers, partnered fathers, CSLC survey

## Abstract

**Background:**

In Japan, ten percent of single-parent households are led by fathers. Taking care of children as a single father is very stressful and could put a strain on their health. It is very important to prevent and identify psychological distress among fathers for both their own health and to avoid negative impacts on children. This study aims to determine the prevalence of and factors associated with psychological distress among single fathers and understand how it is different from partnered fathers.

**Methods:**

We used data from the Comprehensive Survey of Living Conditions 2016. Psychological distress, assessed using the K6 scale, was analyzed among 868 single and 43,880 partnered fathers. Logistic regression analysis was performed to assess the risk factors for psychological distress, such as employment type, sleep hours, and smoking and drinking habits.

**Results:**

Single fathers had a higher proportion (8.5%) of psychological distress compared to partnered fathers (5.0%). A larger percentage of single fathers had a lower educational level and were more likely to be non-regular workers, self-employed, or unemployed than partnered fathers. Among single fathers, the crude and adjusted odds ratio for employment type and sleep hours were significantly associated with psychological distress.

**Conclusion:**

As single parents who are self-employed or directors are likely to have significantly reduced psychological distress than those with regular jobs, measures are needed to improve the work-family balance for non-self-employed fathers. There is a need to provide greater financial assistance and other social welfare support to single parents to ensure their and their children’s good health.

## INTRODUCTION

Single parenting is prevalent all over the world, and the proportion of single parents is increasing with the increase in rates of separation, divorce, and premarital childbearing.^1^ Men nowadays are expected to be involved during pregnancy, birth, and child-rearing activities, in contrast to previous generations. This trend can also be observed in Japan, where the average size of a household decreased by a third from 1970, to 2.4 individuals in 2010.^2^ Additionally, the proportion of single-parent households increased from 5.7% to 8.7% from 1970 to 2010,^2^ with the increasing rates of divorce.^3^ Ten percent of single-parent households in Japan are led by fathers.^4^^,^^5^ These households have often low-income, and the average annual income of single fathers is only 80% of the equivalized average income of two-parent households.^6^ The physical and mental health of single fathers has not received as much attention as that of single mothers, who are more common.^7^ Single fathers have been eligible to receive subsidies for rearing their children since 2010, yet they often still face financial difficulties.^8^ Taking care of children as a single father is very stressful and could deteriorate their health.

Studies suggest that single parents are more likely to have poor mental and physical health than partnered parents.^7^^,^^9^ Studies from Canada, the United Kingdom, and South Korea that compared single and partnered fathers showed that single fathers have poorer self-rated health,^10^ more depression,^11^ and poorer quality of life.^12^ This makes single fathers likely to commit suicide in a country like Japan, where suicide rates have remained high for a long time.^13^^,^^14^ In comparison to partnered fathers, single fathers have less social support and are more likely to smoke tobacco and drink, which is associated with three-fold higher mortality rates in single fathers compared to partnered fathers and single mothers.^15^ This worse health condition is associated with single fathers’ lower socio-economic condition.^7^

Depression among single mothers and its effect on child development is well known.^16^ A growing body of research suggests that paternal depression elevates the prevalence of childhood depression,^17^ as it is associated with unwillingness to involve in child-rearing activities.^16^ Depressed single fathers have been found to use physical disciple on children more often than non-depressed fathers.^18^^,^^19^ Thus, it is very crucial to prevent and identify depression among single fathers to avoid negative impacts on both them and their children. Attention needs to be given to such single fathers, as people with mental illness experience many preventable diseases, such as diabetes, coronary heart disease, and metabolic syndrome.^20^^,^^21^ Family support services were started in Japan in 2009, as per the Child Welfare Act.^22^ However, men are often reluctant to access healthcare services. Compared to single mothers, they are more often found to report poor self-rated mental and physical health but are less likely to seek medical help.^23^ A study found that 80% of the men refused to seek medical help until encouraged by their partners.^24^

Growing evidence in the medical literature has identified unemployment, age greater than 30 years at 6 weeks after childbirth,^25^ financial instability, older age, work-life balance, and relationship issues with children as risk factors for paternal depression.^26^^,^^27^ To date, not many studies focusing on the wellbeing of single fathers in Japan have been published, and a few emerging studies have shown that single fatherhood is related to levels of psychological distress at elevated levels similar to those of single mothers.^28^ These studies, however, focused on the postnatal period or did not use nationally representative data. It remains unclear what factors are associated with psychological distress and how it differs in single and partnered fathers. It is vital to understand what factors contribute to the development of psychological distress among single fathers, and the distinction of these factors from partnered fathers is crucial to deliver assistance to single fathers. Hence, this study aims to determine the prevalence of and factors associated with psychological distress among single fathers and understand how it is different from partnered fathers.

## METHODS

### Data

In June and July 2016, the 11^th^ large-scale Comprehensive Survey of Living Conditions (CSLC) was conducted nationwide by the Ministry of Health, Labour and Welfare of Japan, collecting data on household, health, income, and living conditions.^29^ In this national cross-sectional survey, data from a self-administered household and health survey was used. The questionnaires were distributed to approximately 710,000 people in 289,470 households from 5,410 randomly selected areas from the 2010 National Census. Members from 224,641 households completed the questionnaire (household-level response rate of 77.6%). We obtained permission from the Ministry of Health, Labour and Welfare of Japan to use the data for the intended purpose of this study. We also obtained approval from the ethics committee at the National Centre for Child Health and Development (No. 2020-299).

**Figure 1.**
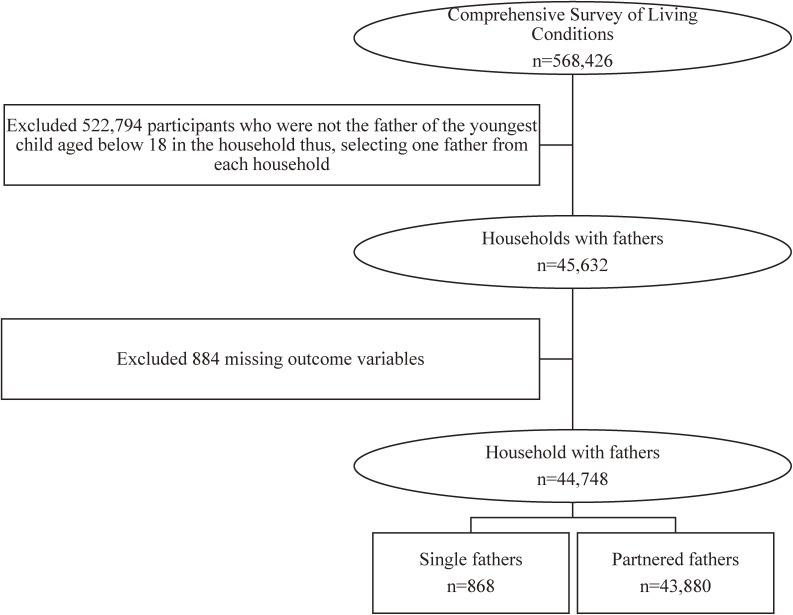
Flow chart of the data extraction process

For the current study, we merged data from the household and health survey questionnaire. We prepared a dataset with data from 568,426 respondents living in 224,208 households. Figure 1 shows how we extracted the study population selecting one father, single or partnered, from each household using the defined inclusion and exclusion criteria. The final sample size consisted of 868 single and 43,880 partnered fathers aged 18 years and above, who had a child aged below 18 years. Table 1 shows the characteristics of the study participants in detail.

**Table 1.** Characteristics of single and partnered fathers, their children and household

	Single		Partnered		
Variables	Frequency (*N* = 868)	Per cent (%)	Frequency (*N* = 43,880)	Per cent (%)	*P*-value
FATHER					
Age, years, mean (SD)	43.3 (8.7)	—	42.0 (7.7)	—	<0.01
Age category, years					<0.01
18–34	117	13.5	7,651	17.4	
35–44	373	43.0	19,928	45.4	
45–54	303	34.9	13,987	31.9	
≥55	75	8.6	2,314	5.3	
Marital Status					NA
Married	—	—	43,880	100.0	
Unmarried	67	7.7	—	—	
Widow	132	15.2	—	—	
Divorced	669	77.1	—	—	
Education					<0.01
High school or less	449	51.7	17,311	39.5	
Vocational school/Jr/Technical College	139	16.0	6,414	14.6	
University or graduate school	149	17.2	15,194	34.6	
Missing	131	15.1	4,961	11.3	
Employment type					<0.01
Regular	538	62.0	33,873	77.2	
Non-regular	72	8.3	1,429	3.3	
Self-employed or director	183	21.1	7,372	16.8	
Unemployed	52	6.0	602	1.4	
Missing	23	2.7	604	1.4	
Work hours^a^					<0.01
≤39 hours	94	12.5	2,883	7.1	
40–55 hours	546	72.5	27,675	68.3	
≥56 hours	113	15.0	9,982	24.6	
Outpatient visit except for depression					<0.05
No	659	75.9	33,993	77.5	
Yes	198	22.8	9,650	22.0	
Missing	11	1.3	237	0.5	
Undergone medical exam during the past year					<0.01
Yes	612	70.5	36,743	83.7	
No	243	28.0	6,667	15.2	
Missing	13	1.5	470	1.1	
Sleep hours					0.8
<6 hours	363	41.8	18,555	42.3	
6 or more	500	57.6	24,999	57.0	
Missing	5	0.6	326	0.7	
Drinking habit					<0.01
Drinkers	490	56.4	28,537	65.0	
Non-drinkers	361	41.6	14,797	33.7	
Missing	17	2.0	546	1.2	
Smoking habit					<0.01
Smokers	465	53.6	17,651	40.2	
Non-smokers	383	44.1	25,600	58.3	
Missing	20	2.3	629	1.4	
Psychological distress					<0.01
Absent	794	91.5	41,689	95.0	
Present	74	8.5	2,191	5.0	
HOUSEHOLD					
Type of home					<0.01
Own home	680	78.3	32,463	74.0	
Rented room or other	188	21.7	11,417	26.0	
Family type					<0.01
Nuclear	370	42.6	35,638	81.2	
Three generation or other households	498	57.4	8,242	18.8	
Household expenditure per person per month					<0.01
<50 thousand	198	22.8	11,115	25.3	
50–74 thousand	322	37.1	16,439	37.5	
75–99 thousand	138	15.9	8,157	18.6	
≥100 thousand	170	19.6	6,403	14.6	
Missing	40	4.6	1,766	4.0	
CHILD					
Age of youngest child, years, mean (SD)	11.7 (4.3)	—	7.7 (5.4)	—	<0.01
Age category of the youngest child					<0.01
0–5 years	94	10.8	18,028	41.1	
6–11 years	269	31.0	12,948	29.5	
12–17 years	505	58.2	12,904	29.4	
Number of children, mean (SD)	1.4 (0.5) (Median: 1)	—	1.6 (0.5) (Median: 2)	—	<0.01
Number of children					<0.01
1 child	553	63.7	18,323	41.8	
2 or more children	315	36.3	25,557	58.2	

SD, standard deviation.

^a^For these variable questions were asked as a secondary question and thus the numbers do not add up to *N*.

### Measures

#### Classification of single and partnered fathers

In this study, fathers were classified as single if their household consisted of fathers, who were living with just children or living with children in a three-generation household and defined as partnered if they were married fathers living in a household of a couple and unmarried child, or a three-generation household with children.

#### Outcome variable

Psychological distress was assessed using the six-item Kessler Screening Scale for Psychological Distress (K6) scale.^30^^,^^31^ The K6 scale has been tested for reliability and validity after translating into Japanese.^31^ For this study, we obtained a Cronbach’s alpha of 0.93 for the K6 scale. This demonstrates an excellent internal consistency and reliability. The K6 scale contains questions starting with “During the last 1 month, how often did you feel” and consists of six related questions about feeling nervous, hopeless, restless, depressed, and worthless. Respondents self-report as 0 (“none of the time”) to 4 (“all of the time”) with a five-point response, the sum of the scores ranging from 0 to 24. Calibration studies have identified a total score of 0–7, 8–12, and 13–24 to represent low, moderate, and high-likelihood of psychological distress, respectively.^30^^,^^32^^,^^33^ For the current study, a total K6 score of 0–12 was classified as without psychological distress and a score of 13 and above as having psychological distress according to the recommended cut-off points.^32^^,^^34^^,^^35^

#### Explanatory variables

We chose employment type, working hours, outpatient department (OPD) visit except for depression, medical exam during the past year, sleep hours, smoking and drinking habits, type of home, family type, household expenditure per person per month, age, education, child’s age, and the number of children as explanatory variables based on findings from previous studies.^26^^,^^27^^,^^33^^,^^36^^,^^37^ Due to the low number of observations in each original variable category for single fathers, most of them have been recategorized into fewer categories. Further details on the re-categorization of explanatory variables can be obtained from eMaterials 1.

### Statistical analysis

The proportions of participants, overall as well as separately for single and partnered fathers, were calculated for all variables of interest. Similarly, the proportions of participants with psychological distress were calculated separately for single and partnered fathers for all variables. Data are presented as percentages. We used fisher’s exact test or chi-square test to compare categorical variables and obtained the *P*-value to examine the difference in the proportion of variable categories for presence and absence of psychological distress. We used logistic regression analysis with listwise deletion for missing observations to estimate the odds ratios (ORs) and 95% confidence intervals (CIs). We initially conducted a bivariate analysis, without any adjustment. Then we applied a logistic regression model for the outcome variable psychological distress and adjusted for education, employment, OPD visit except for depression, medical examination during the past year, sleep hours, drinking and smoking habits, type of home, family type, household expenditure per person per month, age category of youngest child, and number of children. Father’s age was not included in the final model, as it was collinear with the child’s age as determined using Pearson’s correlation coefficient of 0.6. Each analysis was conducted separately for single and partnered fathers. With increasing age as children enter different phases of adolescence, the varying social context surrounding children may have had some contextual effects^37^ on the psychological distress among single fathers. The explanatory variables could be differently associated with psychological distress depending on whether the child is in the preschool age (0–5 years old), elementary school age (6–11 years old), or adolescent age (12–17 years old). To understand the situation better, we analyzed the descriptive statistics of single fathers separately by child’s age (eTable 1). All analyses were conducted using Stata MP 14.2 (Stata Corp, College Station, TX, USA).

## RESULTS

Table 1 shows the descriptive statistics for single and partnered fathers. The distribution of most of the variables was different between single and partnered fathers. Single fathers had significantly higher proportion of psychological distress (8.5%) compared to partnered fathers (5.0%). Single fathers were older and were more likely to have an educational level of high school or less compared to partnered fathers. A higher proportion of single fathers were non-regular workers, self-employed, or unemployed than partnered fathers. Single fathers were less likely to have undergone medical examination during the past year and were less likely to drink but more likely to smoke compared to partnered fathers. More single fathers lived in their own home and in a three-generation or other household compared to partnered fathers.

Table 2 shows those with and without psychological distress by demographic characteristics separately for single and partnered fathers. The distribution of father’s employment type and sleep hours were significantly different between those with and without psychological distress among single fathers. In the case of partnered fathers, all variables except family type, household expenditure per person per month, age, and number of children had significantly different distribution among those with and without psychological distress. The chi-square test could not be applied to the marital status variable, as all the partnered fathers were married.

**Table 2.** Characteristics of single and partnered fathers with and without psychological distress

Variables	Single fathers			Partnered fathers		
	No psychological distress (*N* = 639)	Psychological distress (*N* = 47)	*P*-value^a^	No psychological distress (*N* = 35,452)	Psychological distress (*N* = 1,470)	*P*-value^a^
	*n* (%)	*n* (%)		*n* (%)	*n* (%)	
FATHER						
Age, years, mean (SD)	43.2 (8.2)	44.5 (8.2)	0.2	42.0 (7.6)	41.5 (7.6)	<0.01
Age category, years			0.4			<0.05
18–34	85 (13.3)	5 (10.6)		6,127 (17.3)	289 (19.7)	
35–44	283 (44.3)	17 (36.2)		16,146 (45.5)	672 (45.7)	
≥45	271 (42.4)	25 (53.2)		13,179 (37.2)	509 (34.6)	
Marital status			1.0^b^			—
Married	—	—		35,452 (100)	1,470 (100)	
Unmarried	43 (6.7)	3 (6.4)		—	—	
Widow	93 (14.6)	7 (14.9)		—	—	
Divorced	503 (78.7)	37 (78.7)		—	—	
Education			0.9			<0.01
High school or less	389 (60.9)	29 (61.7)		15,608 (44)	711 (48.4)	
Higher degree	250 (39.1)	18 (38.3)		19,844 (56)	759 (51.6)	
Employment type			<0.01			<0.01
Regular	413 (64.6)	28 (59.6)		28,058 (79.1)	1,117 (76)	
Non-regular	50 (7.8)	8 (17)		1,136 (3.2)	65 (4.4)	
Self-employed or director	143 (22.4)	5 (10.6)		5,833 (16.5)	228 (15.5)	
Unemployed	33 (5.2)	6 (12.8)		425 (1.2)	60 (4.1)	
Work hours^c^			0.8			<0.01
≤39 hours	72 (12.1)	6 (15.8)		2,352 (6.9)	126 (9.3)	
40–55 hours	438 (73.7)	27 (71.1)		23,452 (68.7)	827 (60.9)	
≥56 hours	84 (14.1)	5 (13.2)		8,320 (24.4)	406 (29.9)	
Outpatient visit except for depression			0.8			<0.01
No	488 (76.4)	35 (74.5)		27,568 (77.8)	1,036 (70.5)	
Yes	151 (23.6)	12 (25.5)		7,884 (22.2)	434 (29.5)	
Undergone medical exam during the past year			0.1			<0.01
Yes	462 (72.3)	28 (59.6)		30,350 (85.6)	1,164 (79.2)	
No	177 (27.7)	19 (40.4)		5,102 (14.4)	306 (20.8)	
Sleep hours			<0.05			<0.01
<6 hours	257 (40.2)	27 (57.4)		14,851 (41.9)	848 (57.7)	
6 hours or more	382 (59.8)	20 (42.6)		20,601 (58.1)	622 (42.3)	
Drinking habit			0.6			<0.01
Drinkers	362 (56.7)	25 (53.2)		23,549 (66.4)	881 (59.9)	
Non-drinkers	277 (43.3)	22 (46.8)		11,903 (33.6)	589 (40.1)	
Smoking habit			0.1			<0.05
Smokers	349 (54.6)	32 (68.1)		14,104 (39.8)	634 (43.1)	
Non-smokers	290 (45.4)	15 (31.9)		21,348 (60.2)	836 (56.9)	
HOUSEHOLD						
Type of home			0.8			<0.01
Own home	512 (80.1)	39 (83.0)		26,477 (74.7)	1,020 (69.4)	
Rented or other	127 (19.9)	8 (17.0)		8,975 (25.3)	450 (30.6)	
Family type			0.4			0.1
Nuclear	264 (41.3)	23 (48.9)		28,900 (81.5)	1,226 (83.4)	
Three generation or other households	375 (58.7)	24 (51.1)		6,552 (18.5)	244 (16.6)	
Household expenditure per person per month			0.9			0.2
<50 thousand	148 (23.2)	9 (19.1)		9,280 (26.2)	349 (23.7)	
50–74 thousand	259 (40.5)	21 (44.7)		13,876 (39.1)	590 (40.1)	
75–99 thousand	102 (16.0)	8 (17.0)		6,882 (19.4)	296 (20.1)	
≥100 thousand	130 (20.3)	9 (19.1)		5,414 (15.3)	235 (16)	
CHILD						
Age of youngest child, years, mean (SD)	11.8 (4.1)	12.1 (4.2)	0.7	7.6 (5.4)	7.4 (5.3)	0.06
Age category of the youngest child			0.8			0.1
0–5 years	62 (9.7)	5 (10.6)		14,641 (41.3)	642 (43.7)	
6–11 years	196 (30.7)	12 (25.5)		10,416 (29.4)	431 (29.3)	
12–17 years	381 (59.6)	30 (63.8)		10,395 (29.3)	397 (27)	
Number of children			0.7			0.2
1 child	410 (64.2)	29 (61.7)		14,872 (41.9)	642 (43.7)	
2 or more children	229 (35.8)	18 (38.3)		20,580 (58.1)	828 (56.3)	

SD, standard deviation.

^a^*P*-value of the difference between with and without psychological distress was calculated using the chi-square test or fisher’s exact test for categorical variables and *t*-test for continuous variables.

^b^*P*-value obtained from fisher’s exact test.

^c^Total number of observations does not sum to *N* as it was asked as a sub-question.

Table 3 shows the results of logistic regression analysis among single fathers. Employment type and sleep hours were significantly associated with psychological distress. In the crude analysis, unemployed single fathers had significantly higher odds of psychological distress than those with regular employment (OR 3.42; 95% CI, 1.58–7.39). However, the adjusted odds ratio indicated that the association between any unemployment and psychological distress among single fathers was no longer statistically significant (OR 2.19; 95% CI, 0.74–6.50). Single fathers who were self-employed had significantly lower odds of psychological distress compared to regular employees, even after adjustment for other explanatory variables (OR 0.33; 95% CI, 0.12–0.96). For both crude and adjusted analysis, single fathers who slept less than 6 hours had significantly increased odds of psychological distress (crude OR 2.01; 95% CI, 1.22–3.31 and adjusted OR 2.02; 95% CI, 1.08–3.79) compared to those who slept 6 hours or more. The association between other factors and psychological distress among single fathers was not statistically significant.

**Table 3.** Associated factors of psychological distress among single fathers

	Single fathers			
Variables	Crude OR	95% CI	Adjusted OR	95% CI
Education				
High school or less	Reference			
Higher degree	0.98	(0.56–1.70)	1.05	(0.54–2.02)
Employment type				
Regular	Reference			
Non-regular	2.05	(0.94–4.47)	2.10	(0.85–5.22)
Self-employed or director	0.92	(0.46–1.85)	0.33	(0.12–0.96)
Unemployed	3.42	(1.58–7.39)	2.19	(0.74–6.50)
Outpatient visit except for depression				
Yes	1.37	(0.79–2.36)	1.09	(0.52–2.27)
Medical exam during the past year				
No	1.59	(0.95–2.64)	2.03	(0.98–4.24)
Sleep hour				
Less than 6 hours	2.01	(1.22–3.31)	2.02	(1.08–3.79)
Drink				
Non-drinkers	1.35	(0.83–2.21)	1.12	(0.60–2.10)
Smoke				
Smokers	1.46	(0.87–2.44)	1.74	(0.89–3.42)
Type of home				
Own home	Reference			
Rented	1.18	(0.68–2.06)	0.45	(0.19–1.09)
Type of family				
Nuclear	Reference			
Three generation or other households	0.68	(0.42–1.09)	0.63	(0.32–1.21)
Household expenditure per person per month				
<50 thousand yen	1.30	(0.68–2.48)	0.78	(0.33–1.82)
50–74 thousand yen	Reference			
75–99 thousand yen	1.24	(0.60–2.56)	1.07	(0.44–2.61)
≥100 thousand yen	1.17	(0.58–2.33)	1.06	(0.44–2.59)
Age category of the youngest child				
0–5 years	1.10	(0.47–2.57)	1.50	(0.49–4.63)
6–11 years	Reference			
12–17 years	1.16	(0.67–1.98)	1.69	(0.79–3.63)
Number of children				
1 child	Reference			
2 or more	0.95	(0.58–1.56)	1.50	(0.75–3.03)

CI, confidence interval; OR, odds ratio.

Similarly, eTable 2 shows that single fathers who were unemployment, had a history of OPD visit except for depression, had an absence of medical examination during the past year, were non-drinkers, and who were living in a rented home had significantly higher odds of psychological distress than their respective reference groups among partnered fathers. Non-regular workers and unemployed fathers had significantly higher crude odds of psychological distress than regular working partnered fathers. The association for non-regular employment type, however, was not significant after adjustment with covariates.

eTable 1 shows the distribution of variables by the child’s age category among single fathers. A quarter of fathers of children in the youngest age group were unmarried. A large proportion of fathers with a child aged 12–17 years were self-employed or directors. Fathers of children aged 6–11 years had the lowest proportion (7.8%) of psychological distress compared to fathers of children aged 0–5 years (8.5%) and 12–17 years (8.9%).

## DISCUSSION

We found that Japanese single fathers had a higher prevalence of psychological distress compared to partnered fathers. Sleep hours and employment type had a significant association with psychological distress among single fathers. A low proportion of single fathers had a higher educational degree or had undergone medical examination during the past year and were more likely to be unemployed or non-regularly employed or smoke, which was different from partnered fathers.

Our findings showed that the prevalence of psychological distress among single fathers is 8.5%, while that among partnered fathers is 5%. This difference in prevalence is consistent with the findings of Tobias et al^9^ in a study of New Zealander fathers and Cooper et al^11^ in a United Kingdom study. A study on single mothers found psychological distress among 11% of non-cohabiting single mothers.^36^ Studies have reported that marriage has a protective effect on men’s mental health.^38^^–^^40^ In our study, a large proportion of single fathers were divorced or separated from their spouses. Around 50% of single fathers lived in three-generation or other households. Amidst a shortage of suitable childcare facilities and lack of flexible working schedules in regular jobs, single fathers live in a three-generation household to provide better care for their children.^41^ An additional helping hand might relieve the stress of raising a child alone. However, studies have shown that the stay of single parents in three-generation households could be short-lived with frequent transitions.^42^ Hence, single parents may end up being solely responsible for taking care of their children, as they lack social and mental support.^43^ Additionally, having fewer people to trust, depend on, or make connections with may have considerably affected the prevalence of psychological distress among single fathers.^7^^,^^9^^,^^10^^,^^44^ The pathway to single parenthood, especially spousal conflict during and after separation, might affect stress levels and the simultaneous coping mechanisms leading to varying mental health outcomes.^45^

This study found both increased crude and adjusted odds of psychological distress among unemployed single fathers and decreased odds among self-employed fathers compared to fathers in regular employment. This may be because being unemployed, besides having sole-parenting roles and responsibilities, is a dual burden for single fathers, with an increase in stress.^46^ Furthermore, this may be due to the lack of work-life flexibility in full-time regular employment in Japan. The high prevalence of non-regular employment among single fathers is mostly linked to lower-income and social security benefits.^3^ With less financial support from the government, single fathers may encounter an increased stress level and low social support for coping.

Our findings showed that sleep of less than 6 hours is associated with increased psychological distress. It is an established fact that sleep plays an important role in mental health balance.^47^ Single fathers with young children often juggle lack of sleep and often complain about sleeplessness.^48^ Several studies have shown loneliness to be associated with deteriorating health and is also responsible for interrupted sleep.^49^ With constant pressure to keep up the financial status of the household, single fathers may have sleep deprivation, which may lead to accumulation of stress. Conversely, psychological distress could have led to poor sleep among these parents.^27^ However, we could not establish a causal relationship between sleep and psychological distress because the data we used were cross-sectional.

Our study showed similar findings to the study by Raymo et al, which indicated that single parents tend to have a lower educational level compared to partnered parents.^50^ Similarly, a large number of single fathers had non-regular jobs or were unemployed and were more likely to work fewer hours than partnered fathers. Single fathers were also less likely to have undergone a medical examination in the past year and also less likely to consult regarding their stress compared to partnered fathers.^23^^,^^24^ This suggests a living environment of unstable employment, reduced income, higher stress, and lower levels of health-seeking behavior than partnered fathers, although we did not obtain significant association except for employment. This pattern is particularly concerning given the potentially higher prevalence of key risk factors that we identified.

Of special importance in this context is single fathers’ tobacco use. Although we found single fathers are less likely to drink than partnered fathers, we found that 53.6% of single fathers consume cigarettes regularly compared to 40.2% of partnered fathers, which is consistent with the findings of Chiu et al.^15^ Prevalence of everyday smoking is high (32%) even among Japanese single mothers living in two-generation households.^36^ Studies have confirmed that single parents spend 10–20% of the household income per person on tobacco products.^51^^,^^52^ The high prevalence of smoking among both single and partnered fathers is concerning and suggests an urgent need to implement programs targeting fathers to reduce nicotine abuse and to further strengthen Japan’s tobacco control policies in general. Second-hand smoke exposure could affect their children’s health and may be associated with future increased risk of tobacco consumption among these children.^53^

A quarter of single fathers of children aged 12–17 years were self-employed or directors. These fathers themselves are more likely to be older than fathers of younger children. Thus, these older fathers are more likely to hold the position of a director following the seniority-based wage system in Japan.^54^ Our findings showed that a higher proportion of fathers of children aged 0–5 and 12–17 years had psychological distress than fathers of 6–11-year-olds. As more than half of the fathers of children in the youngest age group were young themselves, they might find parenting stressful.^55^ Similarly, as children enter adolescence, they desire autonomy and experience mood swings, which could lead to psychological distress in their fathers.^56^

The identified risk factors of psychological distress from this study could be used to identify single fathers at higher risk of psychological distress. Our result shows that single fathers have a higher prevalence of psychological distress compared to partnered fathers, suggesting a possibility of an increase in irregularly employed single fathers with psychological distress. Future policies that aim to reduce mental health problems, including psychological distress, should also focus on single-father households, as they have numerous existing disadvantages. Strategies and policies need to be formulated to help recognize and mitigate behavioral and lifestyle risk factors among single fathers for both their own wellbeing and that of their children. However, such strategies will be ineffective while single fathers continue to face economic challenges that make healthy lifestyle choices more difficult. In Japan, although educational, financial, and other support have been prioritized for single parents, these measures do not seem enough. According to a Ministry of Health, Labour and Welfare report, only 8% of single-father households received welfare payments,^57^ suggesting that welfare support is not properly utilized by the small number of single fathers in Japan. There is a need to loosen the eligibility standard for receiving welfare and increase the benefit amount for single-parent families. Free childcare programs have been initiated in Japan since October 2019; however, concerns are being raised over the rapidly increasing number and length of waitlists, as except for low-income families, parents with full-time regular employment are prioritized over parents with non-regular employment.^58^^,^^59^ Childcare centers should be expanded under high priority with improved day-care environments to provide children with the best care possible. These improved services would help reduce the stress and anxiety of single fathers and ensure that they maximize their efforts caring for their children. Increased funding and support for the childcare system generally, and expanding the pool of childcare workers, will likely benefit single fathers and should be considered.

To the best of our knowledge, this is the first study that describes the prevalence and risk factors of psychological distress among single fathers and compares it with partnered fathers using a nationally representative data. This study, however, has a few limitations that need to be considered. Although this was a nationally representative study, it only included a small number of single fathers. A study with a larger number of single fathers would help us understand the risk factors better. Additionally, as we utilized cross-sectional data, we could not conclude the direction of causality in the relationship of psychological distress with various employment and behavioral changes, such as sleep hours and smoking habits. Similarly, although this study had a response rate of 77.6%, missing observations might have resulted in selection bias, thus affecting the results of this study. Moreover, the K6 variable that we used as our outcome variable was acquired using a self-administered questionnaire and not through a structured interview. In addition, one should be cautious when generalizing the findings of this study to other countries given the difference in associated cultural values, the structure of the household, and perceived parental roles. Moreover, in Japan after divorce, there is a high probability that a young child gets to stay with the mother. Therefore, we did not have enough data, especially for single fathers with children aged 0–5 years, to do a subgroup analysis by child’s age.

### Conclusion

In conclusion, the prevalence of psychological distress in single fathers and partnered fathers was 8.5% and 5%, respectively. Single fathers who were self-employed or directors and who slept more than 6 hours were significantly less likely to report psychological distress. As single parents who are self-employed or directors are likely to have significantly reduced psychological distress compared to those with regular jobs, measures are needed to improve the work-family balance for non-self-employed fathers. Additionally, integrated social and health care services should be provided to these fathers to ensure their wellbeing in society. There is a need to provide greater financial assistance and other social welfare support within a framework of a general package of improved welfare rights for single parents to ensure their good health and their children’s healthy and fulfilling development.

## SUPPLEMENTARY MATERIAL

eMaterials 1. Detailed description on recategorization of explanatory variables

eTable 1. Descriptive characteristics of single father separately by child’s age

eTable 2. Associated factors of psychological distress among partnered fathers

## References

[1] World Family Map 2015. Mapping Family Change and Child Well-Being Outcomes; 2015. Accessed June 15, 2020. https://www.childtrends.org/wp-content/uploads/2015/09/2015-39WorldFamilyMap2015.pdf.

[2] National Institute of Population and Social Security Research. Population statistics of Japan 2012. Published 2012. Accessed June 12, 2020. http://www.ipss.go.jp/index-e.asp.

[3] Kachi Y, Inoue M, Nishikitani M, Yano E. Differences in self-rated health by employment contract and household structure among Japanese employees: a nationwide cross-sectional study. J Occup Health. 2014;56(5):339–346. 10.1539/joh.13-0279-OA 25230825

[4] Ministry of Health Labour and Welfare. National Survey on Single Parent Families in 2016. Published 2016. Accessed June 15, 2020. https://www.mhlw.go.jp/stf/seisakunitsuite/bunya/0000188147.html.

[5] Ministry of Internal Affairs and Communication. Final report of 2015 “Population and Households of Japan.” Statistics Bureau of Japan. Published 2015. Accessed June 15, 2020. https://www.stat.go.jp/english/data/kokusei/2015/final_en/final_en.html#Summary.

[6] Ministry of Health Labour and Welfare of Japan. 2016 National Survey Results Report on Single Parent Households. Published 2016. Accessed July 21, 2021. https://www.mhlw.go.jp/stf/seisakunitsuite/bunya/0000188147.html.

[7] Kong KA, Choi HY, Kim SI. Mental health among single and partnered parents in South Korea. PLoS One. 2017;12(8):e0182943. 10.1371/journal.pone.0182943 28806734 PMC5555686

[8] The Japan Times. Single fathers emerge from the shadows. Published 2014. Accessed June 15, 2020. https://www.japantimes.co.jp/news/2014/08/20/national/social-issues/single-fathers-emerge-shadows/#.Xubffy-cboB.

[9] Tobias M, Gerritsen S, Kokaua J, Templeton R. Psychiatric illness among a nationally representative sample of sole and partnered parents in New Zealand. Aust N Z J Psychiatry. 2009;43(2):136–144. 10.1080/00048670802607196 19153921

[10] Janzen BL, Green K, Muhajarine N. The health of single fathers: demographic, economic and social correlates. Can J Public Health. 2006;97(6):440–444. 10.1007/BF03405224 17203721 PMC6975882

[11] Cooper C, Bebbington PE, Meltzer H, . Depression and common mental disorders in lone parents: results of the 2000 National Psychiatric Morbidity Survey. Psychol Med. 2008;38(3):335–342. 10.1017/S0033291707001493 17892621

[12] Kong KA, Kim SI. Mental health of single fathers living in an urban community in South Korea. Compr Psychiatry. 2015;56:188–197. 10.1016/j.comppsych.2014.09.012 25281990

[13] Dhungel B, Sugai MK, Gilmour S. Trends in suicide mortality by method from 1979 to 2015 in Japan. Int J Environ Res Public Health. 2019;16(10):1794. 10.3390/ijerph16101794 31117173 PMC6571574

[14] Dhungel B, Murakami T, Wada K, Gilmour S. Mortality risks among blue- and white-collar workers: a time series study among Japanese men aged 25–64 years from 1980 to 2015. J Occup Health. 2021;63(1):e12215. 10.1002/1348-9585.12215 33837627 PMC8035635

[15] Chiu M, Rahman F, Vigod S, Lau C, Cairney J, Kurdyak P. Mortality in single fathers compared with single mothers and partnered parents: a population-based cohort study. Lancet Public Heal. 2018;3(3):e115–e123. 10.1016/S2468-2667(18)30003-3 29454821

[16] Paulson JF, Dauber S, Leiferman JA. Individual and combined effects of postpartum depression in mothers and fathers on parenting behavior. Paediatrics. 2006;118(2):659–668. 10.1542/peds.2005-2948 16882821

[17] Ramchandani PG, Stein A, O’Connor TG, Heron J, Murray L, Evans J. Depression in men in the postnatal period and later child psychopathology: a population cohort study. J Am Acad Child Adolesc Psychiatry. 2008;47(4):390–398. 10.1097/CHI.0b013e31816429c2 18388761 PMC2650418

[18] Davis RN, Davis MM, Freed GL, Clark SJ. Fathers’ depression related to positive and negative parenting behaviors with 1-year-old children. Pediatrics. 2011;127(4):612–618. 10.1542/peds.2010-1779 21402627 PMC3387886

[19] Rodriguez Rodriguez CM. Parental discipline and abuse potential affects on child depression, anxiety, and attributions. J Marriage Fam. 2003;65(4):809–817. 10.1111/j.1741-3737.2003.00809.x

[20] Gilmour S, Mai PL, Nguyen P, Dhungel B, Tomizawa M, Nguyen H. Progress towards health for all: time to end discrimination and marginalization. Int J Environ Res Public Health. 2020;17(5):1696. 10.3390/ijerph17051696 32150920 PMC7084917

[21] Osborn DP, Wright CA, Levy G, King MB, Deo R, Nazareth I. Relative risk of diabetes, dyslipidaemia, hypertension and the metabolic syndrome in people with severe mental illnesses: systematic review and metaanalysis. BMC Psychiatry. 2008;8:84. 10.1186/1471-244X-8-84 18817565 PMC2570660

[22] Ministry of Health Labour and Welfare of Japan. Social care based on the interpretation of Article 3-2 of the revised Child Welfare Act [In Japanese]. Published January 13, 2017. Accessed July 20, 2021. https://www.mhlw.go.jp/file/05-Shingikai-11901000-Koyoukintoujidoukateikyoku-Soumuka/0000148763.pdf.

[23] Ringbäck Weitoft G, Burström B, Rosén M. Premature mortality among lone fathers and childless men. Soc Sci Med. 2004;59(7):1449–1459. 10.1016/j.socscimed.2004.01.026 15246173

[24] Goldenberg SL. Status of men’s health in Canada. Can Urol Assoc J. 2014;8(7–8 Suppl 5):S142–S144. 10.5489/cuaj.2308 25243037 PMC4145701

[25] Ballard CG, Davis R, Cullen PC, Mohan RN, Dean C. Prevalence of postnatal psychiatric morbidity in mothers and fathers. Br J Psychiatry. 1994;164(6):782–788. 10.1192/bjp.164.6.782 7952984

[26] Edward KL, Castle D, Mills C, Davis L, Casey J. An integrative review of paternal depression. Am J Men Health. 2015;9(1):26–34. 10.1177/1557988314526614 24626601

[27] Kumar SV, Oliffe JL, Kelly MT. Promoting postpartum mental health in fathers: recommendations for nurse practitioners. Am J Men Health. 2018;12(2):221–228. 10.1177/1557988317744712 29183251 PMC5818130

[28] Chiu M, Rahman F, Kurdyak P, Cairney J, Jembere N, Vigod S. Self-rated health and mental health of lone fathers compared with lone mothers and partnered fathers: a population-based cross-sectional study. J Epidemiol Community Health. 2017;71(5):417–423. 10.1136/jech-2016-208005 27923873

[29] Ministry of Health Labour and Welfare of Japan. Comprehensive Survey of Living Conditions of the People on Health and Welfare. Published 2016. Accessed June 25, 2020. https://www.mhlw.go.jp/english/database/db-hss/cslc.html.

[30] Kessler RC, Andrews G, Colpe LJ, . Short screening scales to monitor population prevalences and trends in non-specific psychological distress. Psychol Med. 2002;32(6):959–976. 10.1017/S0033291702006074 12214795

[31] Furukawa TA, Kawakami N, Saitoh M, . The performance of the Japanese version of the K6 and K10 in the World Mental Health Survey Japan. Int J Methods Psychiatr Res. 2008;17(3):152–158. 10.1002/mpr.257 18763695 PMC6878390

[32] Kessler RC, Barker PR, Colpe LJ, . Screening for serious mental illness in the general population. Arch Gen Psychiatry. 2003;60(2):184–189. 10.1001/archpsyc.60.2.184 12578436

[33] Hilton MF, Whiteford HA. Interacting with the public as a risk factor for employee psychological distress. BMC Public Health. 2010;10(1):435. 10.1186/1471-2458-10-435 20653982 PMC2918558

[34] Kawakami N, Kondo K, Yanagida K, Furukawa T. Mental health research on the preventive measure against suicide in adulthood. In: Ueda S (principal investigator). *Report of the research grant for the implementation of preventive measure based on the current status of suicide from the Ministry of Health, Labour and Welfare, Japan FY 2004*. Tokyo (Japan): Ministry of Health, Labour and Welfare, Japan; 2005;147–156 (in Japanese).

[35] Kawakami N. Manual for investigating the psychological effects of trauma in the general population (February 2015 edition) In: Health, Labor and Welfare Science Research Grant; Understanding information on mental disorders in the disaster area, verifying the effects. Published 2015. Accessed November 2, 2020. http://plaza.umin.ac.jp/heart/pdf/151026.pdf.

[36] Kato T, Takehara K, Suto M, Sampei M, Urayama KY. Psychological distress and living conditions among Japanese single-mothers with preschool-age children: an analysis of 2016 Comprehensive Survey of Living Conditions. J Affect Disord. 2021;286:142–148. 10.1016/j.jad.2021.02.065 33721741

[37] Giallo R, D’Esposito F, Cooklin A, . Psychosocial risk factors associated with fathers’ mental health in the postnatal period: results from a population-based study. Soc Psychiatry Psychiatr Epidemiol. 2013;48(4):563–573. 10.1007/s00127-012-0568-8 22898826

[38] Rendall MS, Weden MM, Favreault MM, Waldron H. The protective effect of marriage for survival: a review and update. Demography. 2011;48(2):481–506. 10.1007/s13524-011-0032-5 21526396

[39] Gove WR. The relationship between sex roles, marital status, and mental illness. Soc Forces. 1972;51(1):34. 10.2307/2576129

[40] Fu R, Noguchi H. Does marriage make us healthier? Inter-country comparative evidence from China, Japan, and Korea. PLoS One. 2016;11(2):e0148990. 10.1371/journal.pone.0148990 26862896 PMC4749249

[41] Raymo JM, Zhou Y. Living arrangements and the well-being of single mothers in Japan. Popul Res Policy Rev. 2012;31(5):727–749. 10.1007/s11113-012-9247-4 23144521 PMC3490400

[42] Pilkauskas NV. Three generation family households: differences by family structure at birth. J Marriage Fam. 2012;74(5):931–943. 10.1111/j.1741-3737.2012.01008.x 24014117 PMC3765068

[43] Kim DS, Jeon GS, Jang SN. Socioeconomic status, social support and self-rated health among lone mothers in South Korea. Int J Public Health. 2010;55(6):551–559. 10.1007/s00038-010-0169-9 20614228

[44] Ravanera Z. Informal networks social capital of fathers: what does the social engagement survey tell us? Soc Indic Res. 2007;83(2):351–373. 10.1007/s11205-006-9053-7

[45] Amato PR. The Consequences of Divorce for Adults and Children. J Marriage Fam. 2000;62(4):1269–1287. 10.1111/j.1741-3737.2000.01269.x

[46] Katsurada E, Sugihara Y. Gender-role identity, attitudes toward marriage, and gender-segregated school backgrounds. Sex Roles. 2002;47(5/6):249–258. 10.1023/A:1021334710431

[47] Freeman D, Sheaves B, Goodwin GM, . The effects of improving sleep on mental health (OASIS): a randomised controlled trial with mediation analysis. Lancet Psychiatry. 2017;4(10):749–758. 10.1016/S2215-0366(17)30328-0 28888927 PMC5614772

[48] Baker D, Mead N, Campbell S. Inequalities in morbidity and consulting behaviour for socially vulnerable groups. Br J Gen Pract. 2002;52(475):124–130. 11885821 PMC1314218

[49] Holt-Lunstad J, Smith TB, Baker M, Harris T, Stephenson D. Loneliness and social isolation as risk factors for mortality: a meta-analytic review. Perspect Psychol Sci. 2015;10(2):227–237. 10.1177/1745691614568352 25910392

[50] Raymo JM, Bumpass L, Iwasawa M. Marital dissolution in Japan: recent trends and patterns. Demogr Res. 2004;11:395–420. 10.4054/DemRes.2004.11.14

[51] Siahpush M. Socioeconomic status and tobacco expenditure among Australian households: results from the 1998–99 Household Expenditure Survey. J Epidemiol Community Health. 2003;57(10):798–801. 10.1136/jech.57.10.798 14573585 PMC1732284

[52] Dorsett R. An econometric analysis of smoking prevalence among lone mothers. J Health Econ. 1999;18(4):429–441. 10.1016/S0167-6296(98)00045-9 10539615

[53] Kandel DB, Griesler PC, Hu MC. Intergenerational patterns of smoking and nicotine dependence among US adolescents. Am J Public Health. 2015;105(11):e63–e72. 10.2105/AJPH.2015.302775 26378847 PMC4605183

[54] Suzuki M, Ito M, Ishida M, Nihei N, Maruyama M. Individualizing Japan: searching for its origin in first modernity. Br J Sociol. 2010;61(3):513–538. 10.1111/j.1468-4446.2010.01324.x 20840430

[55] Mrayan L, Cornish F, Dhungana N, Parfitt B. Transition to parenthood during the transition to modernity in Jordan: new parents’ views on family and healthcare support systems. Appl Nurs Res. 2016;32:139–143. 10.1016/j.apnr.2016.07.002 27969017

[56] Small SA, Eastman G, Cornelius S. Adolescent autonomy and parental stress. J Youth Adolesc. 1988;17(5):377–391. 10.1007/BF01537880 24277665

[57] Ministry of Health Labour and Welfare of Japan. Results of the 2011 National Survey of Mother and Child Households: Attachment 1; 2012. Accessed April 13, 2021. https://www.mhlw.go.jp/stf/houdou/2r9852000002j6es.html.

[58] Ministry of Health Labour and Welfare. Children and Childrearing: For Achievement of the Zero Children Waiting List. Accessed April 13, 2021. https://www.mhlw.go.jp/english/policy/children/children-childrearing/index.html.

[59] Wataru S. Japan’s Free Childcare Program No Panacea for Daycare Waitlists. *Nippon.com*. https://www.nippon.com/en/in-depth/d00489/japan’s-free-childcare-program-no-panacea-for-daycare-waitlists.html. Published June 11, 2019. Accessed April 13, 2021.

