# Supplementary material for: Prevalence and Associated Factors of Psychological Distress Among Single Fathers in Japan
Source: J Epidemiol. 2023 Jun 5;33(6):294–302. doi: 10.2188/jea.JE20210273 (PMC10165216; doi:10.2188/jea.JE20210273)
Supplement: Supplementary file 1 [file je-33-294-s001.pdf]

eMaterials 1. Detailed description on recategorization of explanatory variables

For the household questionnaire, each participant was asked to answer questions about his/her employment status. The employed respondents were asked about their employment type from the options provided and number of working hours in a week as a free-response question. Employment type was classified as regular (regular employee), non-regular employee (part-time employee, arbeit (short time and term), dispatched employee from temporary labour agency, contract employee, entrusted employee and other), self-employed or director, and unemployed. A regular staff/employee is a person termed as general schedule employee or regular member by the company or organisation for which the person is working. They are full-time workers directly hired by their employers and their employment is not limited in duration except for the mandatory retirement age. The number of working hours was categorised as ≤39 hours per week and 40–55 hours per week and ≥56 hours per week based on legal working hours and overtime hours designated by the Labour Standard Law of Japan.

Questions regarding hospital or clinic visit (outpatient department [OPD] visits) for disease or injury was asked with multiple possible options. Based on presence or absence of medical service visit for any specified disease or injury except for depression, it was divided into two categories: yes and no. Similarly, presence or absence of medical examination during the past year, both regular and comprehensive medical check-up was categorised dichotomously. The average sleeping time during the last one month was recategorized into less than 6 hours, and 6 or more hours. Drinking habit per week was categorised as drinkers (every day, 5 to 6 days a week, 3 to 4 days a week, 1 or 2 days a week, and 1 to 3 days a month) and non-drinkers (rarely drink, quit drinking, don't drink/can't drink). Smoking habit was categorised as smokers (smoke every day, few days a week) and non-smokers (not smoked in the past month, do not smoke). Type of home was categorised as 'own' if the respondent owned

the house and ‘rented’ if they lived in condominium, residence for private or public employees, public apartment house or any other rented room. Type of family was categorised as nuclear (household only for married couple & unmarried child, households with single parents and unmarried children only) and 3 generation and other (three generation household, other households). The total amount of expenditure in the last month, in ten-thousand yen, spent by all household members was asked to the respondents as an open-ended question. This amount was divided by the total number of members in the family to calculate the household expenditure per person and was categorised as <50 thousand yen, 50–74 thousand yen, 75–99 thousand yen, and ≥100 thousand yen. Marital status was classified as married, unmarried, widowed and divorced. Education was classified as high school or less (elementary school/junior high school, high school/junior high school under the old system of education) and higher degree (vocational school, college/technical college, university, post-graduate course). Age of youngest child was divided into three categories as 0–5 years, 6–11 years and 12–17 years based on the corresponding schooling level such as pre-school, elementary school, and junior & high school respectively. Similarly, number of children was categorised into two groups: one child and two or more children.

eTable 1. Descriptive characteristics of single father separately by child's age

	0–5 years		6–11 years		12–17 years	
Variables	n (N=94)	Percent (%)	n (N=269)	Percent (%)	n (N=505)	Percent (%)
FATHER						
Age, years, mean (SD)	33.3 (9.8)	-	40.7 (7.2)	-	46.6 (7.2)	-
Age category, years						
18–34	54	57.4	53	19.7	10	2.0
35–44	30	31.9	144	53.5	199	39.4
≥45	10	10.6	72	26.8	296	58.6
Marital Status						
Unmarried	22	23.4	20	7.4	25	5.0
Widow	12	12.8	37	13.8	83	16.4
Divorced	60	63.8	212	78.8	397	78.6
Education						
High school or less	45	47.9	146	54.3	258	51.1
Higher degree	31	33.0	77	28.6	180	35.6
Missing	18	19.1	46	17.1	67	13.3
Employment type						
Regular	58	61.7	176	65.4	304	60.2
Non-regular	15	16.0	22	8.2	35	6.9
Self-employed or director	11	11.7	43	16.0	129	25.5
Unemployed	7	7.4	19	7.1	26	5.1
Missing	3	3.2	9	3.3	11	2.2
Work hour						
≤39 hours	14	17.3	29	12.7	51	11.5
40–55 hours	50	61.7	174	76.3	322	72.5
≥56 hours	17	21.0	25	11.0	71	16.0

OPD visit except for depression						
No	79	84.0	204	75.8	376	74.5
Yes	11	11.7	63	23.4	124	24.6
Missing	4	4.3	2	0.7	5	1.0
Undergone medical exam during past year						
Yes	59	62.8	191	71.0	362	71.7
No	27	28.7	77	28.6	139	27.5
Missing	8	8.5	1	0.4	4	0.8
Sleep hours						
<6 hours	40	42.6	110	40.9	213	42.2
6 or more	53	56.4	158	58.7	289	57.2
Missing	1	1.1	1	0.4	3	0.6
Drinking habit						
Drinkers	41	43.6	146	54.3	303	60.0
Non-drinkers	46	48.9	119	44.2	196	38.8
Missing	7	7.4	4	1.5	6	1.2
Smoking habit						
Smokers	48	51.1	160	59.5	257	50.9
Non-smokers	38	40.4	105	39.0	240	47.5
Missing	8	8.5	4	1.5	8	1.6
Psychological distress						
Absent	86	91.5	248	92.2	460	91.1
Present	8	8.5	21	7.8	45	8.9
HOUSEHOLD						
Type of home						
Own home	68	72.3	210	78.1	402	79.6
Rented room or other	26	27.7	59	21.9	103	20.4
Family type						
Nuclear	31	33.0	117	43.5	222	44.0

Three generation or other households	63	67.0	152	56.5	283	56.0
Household expenditure per person						
<50 thousand	36	38.3	67	24.9	95	18.8
50–74 thousand	33	35.1	111	41.3	178	35.2
75–99 thousand	11	11.7	41	15.2	86	17.0
≥100 thousand	10	10.6	37	13.8	123	24.4
Missing	4	4.3	13	4.8	23	4.6
CHILD						
Age of youngest child, mean (SD)	3.3 (1.6)	-	8.9 (1.6)	-	14.7 (1.7)	-
Number of children, mean (SD)	1.4 (0.5) (Median: 1)	-	1.6 (0.5) (Median: 2)	-	1.2 (0.4) (median: 1)	-
1 child	51	54.3	110	40.9	392	77.6
2 or more children	43	45.7	159	59.1	113	22.4

OPD, outpatient department; SD, standard deviation.

eTable 2. Associated factors of psychological distress among partnered fathers

	Partnered fathers			
Variables	Crude OR	95% CI	Adjusted OR	95% CI
Education				
High school or less	Reference			
Higher degree	0.86	(0.78–0.94)	0.85	(0.76–0.95)
Employment type				
Regular	Reference			
Non-regular	1.47	(1.18–1.82)	1.23	(0.95–1.60)
Self-employed or director	1.12	(1.00–1.26)	0.88	(0.76–1.03)
Unemployed	3.53	(2.79–4.45)	2.84	(2.12–3.80)
OPD visit except depression				
Yes	1.29	(1.17–1.42)	1.47	(1.31–1.65)
Medical exam during past year				
No	1.61	(1.44–1.80)	1.49	(1.29–1.72)
Sleep hour				
6 hours or less	1.85	(1.69–2.04)	1.89	(1.69–2.08)
Drink				
Non-drinkers	1.26	(1.14–1.38)	1.23	(1.10–1.37)
Smoke				
Non-smokers	0.87	(0.79–0.95)	0.89	(0.80–0.99)
Type of home				
Own home	Reference			
Rented	1.28	(1.17–1.41)	1.17	(1.03–1.32)
Type of family				
Nuclear	Reference			
Three generation or other households	0.92	(0.82–1.03)	0.94	(0.81–1.09)
Household expenditure per person				
<50 thousand yen	0.90	(0.80–1.01)	0.86	(0.75–0.99)
50–74 thousand yen	Reference			
75–99 thousand yen	1.00	(0.88–1.13)	1.02	(0.89–1.18)
≥100 thousand yen	1.03	(0.90–1.18)	1.05	(0.90–1.24)
Age category of youngest child				
0–5 years	1.02	(0.92–1.13)	1.08	(0.95–1.22)
6–11 years	Reference			
12–17 years	0.89	(0.79–1.00)	0.86	(0.74–1.00)
Number of children				
1 child	Reference			
2 or more children	0.97	(0.89–1.06)	0.97	(0.86–1.08)

CI, confidence interval; OR, odds ratio.
